# Lifetime cost-effectiveness analysis of first-line dialysis modalities for patients with end-stage renal disease under peritoneal dialysis first policy

**DOI:** 10.1186/s12882-020-1708-0

**Published:** 2020-02-04

**Authors:** Carlos K. H. Wong, Julie Chen, Samuel K. S. Fung, Maggie Mok, Yuk lun Cheng, Irene Kong, Wai Kei Lo, Sing Leung Lui, T. M. Chan, Cindy L. K. Lam

**Affiliations:** 1 Department of Family Medicine and Primary Care, Li Ka Shing Faculty of Medicine, The University of Hong Kong, Rm 1-01, 1/F, Jockey Club Building for Interdisciplinary Research, 5 Sassoon Road, Pokfulam, Hong Kong, China; 2 Bau Institute of Medical and Health Sciences Education, Li Ka Shing Faculty of Medicine, The University of Hong Kong, Hong Kong, China; 3 Department of Medicine and Geriatrics, Princess Margaret Hospital, Hong Kong, China; 4 Division of Nephrology, Department of Medicine, Tung Wah Hospital, Hong Kong, China; 5 Department of Medicine, Alice Ho Miu Ling Nethersole Hospital, Hong Kong, China; 6 Division of Nephrology, Department of Medicine, Li Ka Shing Faculty of Medicine, The University of Hong Kong, Hong Kong, China

**Keywords:** Cost-effectiveness, Economic burden, End-stage renal disease, Peritoneal dialysis first, Dialysis, Nocturnal home haemodialysis

## Abstract

**Background:**

This study aimed to determine the lifetime cost-effectiveness of first-line dialysis modalities for end-stage renal disease (ESRD) patients under the “Peritoneal Dialysis First” policy.

**Methods:**

Lifetime cost-effectiveness analyses from both healthcare provider and societal perspectives were performed using Markov modelling by simulating at age 60. Empirical data on costs and health utility scores collected from our studies were combined with published data on health state transitions and survival data to estimate the lifetime cost, quality-adjusted life-years (QALYs) and cost-effectiveness of three competing dialysis modalities: peritoneal dialysis (PD), hospital-based haemodialysis (HD) and nocturnal home HD.

**Results:**

For cost-effectiveness analysis over a lifetime horizon from the perspective of healthcare provider, hospital-based HD group (lifetime cost USD$142,389; 6.58 QALYs) was dominated by the PD group (USD$76,915; 7.13 QALYs). Home-based HD had the highest effectiveness (8.37 QALYs) but with higher cost (USD$97,917) than the PD group. The incremental cost-effectiveness ratio (ICER) was USD$16,934 per QALY gained for home-based HD over PD. From the societal perspective, the results were similar and the ICER was USD$1195 per QALY gained for home-based HD over PD. Both ICERs fell within the acceptable thresholds. Changes in model parameters via sensitivity analyses had a minimal impact on ICER values.

**Conclusions:**

This study assessed the cost-effectiveness of dialysis modalities and service delivery models for ESRD patients under “Peritoneal Dialysis First” policy. For both healthcare provider and societal perspectives, PD as first-line dialysis modality was cost-saving relative to hospital-based HD, supporting the existing PD First or favoured policy. When compared with PD, Nocturnal home Home-based HD was considered a cost-effective first-line dialysis modality for ESRD patients.

## Background

With the rising global burden of ESRD, there is a real need for effective and cost-effective renal replacement therapy (RRT) among patients with end-stage renal disease (ESRD) [1]. In Hong Kong where the “Peritoneal Dialysis First” policy [2–4] has been implemented over 25 years, the prevalent cases of patients on peritoneal dialysis (PD) was 3–4 times greater than the those of patients on haemodialysis (HD) [5]. Owing to the increased number of ESRD patients on dialysis and onto transplant waiting list, organs from both living or deceased donors for renal transplantation were scarce over the past decade [6]. Dialysis treatment is a pragmatic first-line treatment option for patients with ESRD. PD is offered as the first-line dialysis treatment to incident dialysis patients in public healthcare sector, whereas HD is offered to patients who have medical contraindications for PD. Another alternative available in Hong Kong is the nocturnal home HD program launched in 2006 [7, 8], commencing HD treatment at home. This option is the least popular, consistent with data from United States Renal Data System, in which only 2.7% of prevalent dialysis patients received home HD [9].

Health economic evidence demonstrated that renal transplantation was increasingly favoured approach to save costs while conferring additional benefits in clinical effectiveness [10, 11]. The lack of availability of live or deceased donor organs remains practical challenges to implement renal transplantation at population level. Increasing ratio of PD/HD among incident dialysis patients and offering access to dialysis at home are shown to be effective and cost-effective strategies [10–12]. Implementation of former strategy may not be applicable to ESRD management in Hong Kong due to the “Peritoneal Dialysis First” policy. However, health economic value for improving HD access and switching dialysis treatment venue from in-centre or hospital to home is uncertain. There is therefore a need to conduct an economic evaluation of the cost-effectiveness of available dialysis modalities in managing ESRD patients in the Hong Kong setting which may provide an additional perspective for consideration by other countries with PD first or favoured policy.

The study aims to determine the lifetime cost-effectiveness of three dialysis modalities (PD, hospital-based HD or home-based HD) as first-line treatment for ESRD patients under the “Peritoneal Dialysis First” policy. We hypothesize that the PD strategy is more cost-effective than the hospital-based HD and home-based strategy from health provider’s perspective but the home-based HD strategy may be a cost-effective dialysis strategy from societal perspective.

## Methods

### Study design

This study compared cost-effectiveness of three dialysis modalities as first-line treatment strategies, and evaluated lifetime cost-effectiveness analysis of the hospital-based HD strategy or home-based HD strategy in comparison with the currently preferred PD strategy in Hong Kong.

### Costing analysis

Costing analysis was performed from both the health provider and societal perspectives. A micro-costing and bottom approach was applied in the costing analysis [13]. From the health provider’s perspective, direct medical costs associated with dialysis and its related health service delivery were taken into consideration. Societal costs were the sum of direct medical costs, direct non-medical costs (e.g. patients’ out-of-pocket costs due to self-prescription and transportation), and indirect costs (e.g. time costs spent by patients and their caregivers). Unit costs of each item were referenced to the Hospital Authority Government Gazette list of health service charges for non-Hong Kong residents in 2017 [14] and the Hospital Authority list of charges for private services in 2017 [15]. Estimation of costs accrued by pre-dialysis surgery, surgery related to the removal of catheter or vascular access, dialysis training and re-training, machine rental and consumables, renal transplantation and post-transplant follow-up, outpatient visits, emergency visits, and hospitalization were entailed in our prior publication [13]. Full list of unit cost of health service items is listed in Table 1.

**Table 1:** Unit cost of each healthcare service related to patients with end-stage renal disease

Healthcare service	Cost (USD)	Reference
General outpatient clinic (per visit)	57.1	Hospital Authority [14]
Specialist outpatient clinic (per visit)	152.6	Hospital Authority [14]
Accident and emergency (per visit)	157.7	Hospital Authority [14]
Hospital stay (per night)	653.8	Hospital Authority [14]
Haemodialysis (per session)	384.6	Hospital Authority [14]
PD home machine rental consumables, maintenance and insurance (per month)	641.0	Personal communication with nephrologists
Home-based HD machine rental and consumables (per month)	769.2	Personal communication with nephrologists
Pre-dialysis access surgery		
PD: insertion / removal of peritoneal dialysis catheter	3192.3	Hospital Authority [15]
HD: Insertion / removal of haemodialysis catheter ^b^	2057.7	Hospital Authority [15]
HD: Arteriovenostomy for renal dialysis (arteriovenous fistula, arteriovenous graft) ^b^	6384.6	Hospital Authority [15]. Personal communication with nephrologists
Renal transplantation	10,278.8	Hospital Authority [15]
Post-transplant follow-up^c^		Personal communication with nephrologists
Initial year	1678.2	
Subsequent years	610.3	
Dialysis training^a^		Personal communication with nephrologists
PD^d^	1692.3	
Hospital-based HD	0	
Home-based HD	12,184.6	
Re-training after peritonitis^a,d^		Personal communication with nephrologists
PD	1692.3	

^a^Dialysis training cost for PD = USD42.3 × 8 h × 5 days × 1 week = USD1392.3; Hospital-based HD = 0; Dialysis training cost for Home-based HD = USD42.3 × 8 h × 3 days × 12 weeks = USD12184.6.

(Median hourly wage of Registered Nurse in 2017 = USD42.3)

^b^The ratio for HD: Insertion / removal of haemodialysis catheter vs. Arteriovenostomy for renal dialysis (arteriovenous fistula, arteriovenous graft) = 3:7, with reference to recommendation by nephrologists

^c^ Post-transplant patients in initial year were assumed to attend eleven times at specialist outpatient clinics (USD152.6 × 11 times = USD1678.2). Post-transplant patients in second years were assumed to attend four times at specialist outpatient clinics (USD152.6 × 4 times = USD610.3)

^d^PD patients are required for re-training after every 5 years for PD life extension

### Health utility measurement

Long-term cost-effectiveness analysis in terms of QALYs required the use of utility scores, estimated from a previously published systematic review [16], and previous HRQOL surveys conducted by our research team [17, 18]. Utility score of renal transplanted patients was taken from the same systematic review [16]. Comparison of SF-12 scores across three dialysis modalities was performed using pooled data from the current study and a previous study conducted by our research team [17, 18]. The results of these HRQOL data measured by SF-12 Health Survey, shown to be valid and reliable in Hong Kong [19], were converted to SF-6D utility scores through Hong Kong Chinese population scoring algorithm [20]. The SF-6D utility scores of ESRD undergoing PD, hospital-based HD and home-based HD patients were further converted to disutility (one minus utility) associated with each health state.

### Cost-effectiveness analysis

Cost-effectiveness analysis was performed by using Markov modelling. Empirical data on costing information and health utility scores taken from our previous studies [13, 18] was combined with published data on utility scores [16, 17], health state transitions [21], transplantation and mortality rates [5, 22, 23] to estimate long-term effectiveness and cost-effectiveness.

### Outcome measures

Outcomes were total costs and total effectiveness quantified by QALYs of three competing dialysis strategies for ESRD management, and incremental cost-effectiveness ratio of hospital-based HD and home-based HD relative to PD strategy from both the healthcare provider and societal perspectives.

### Data analysis

**Fig. 1:**
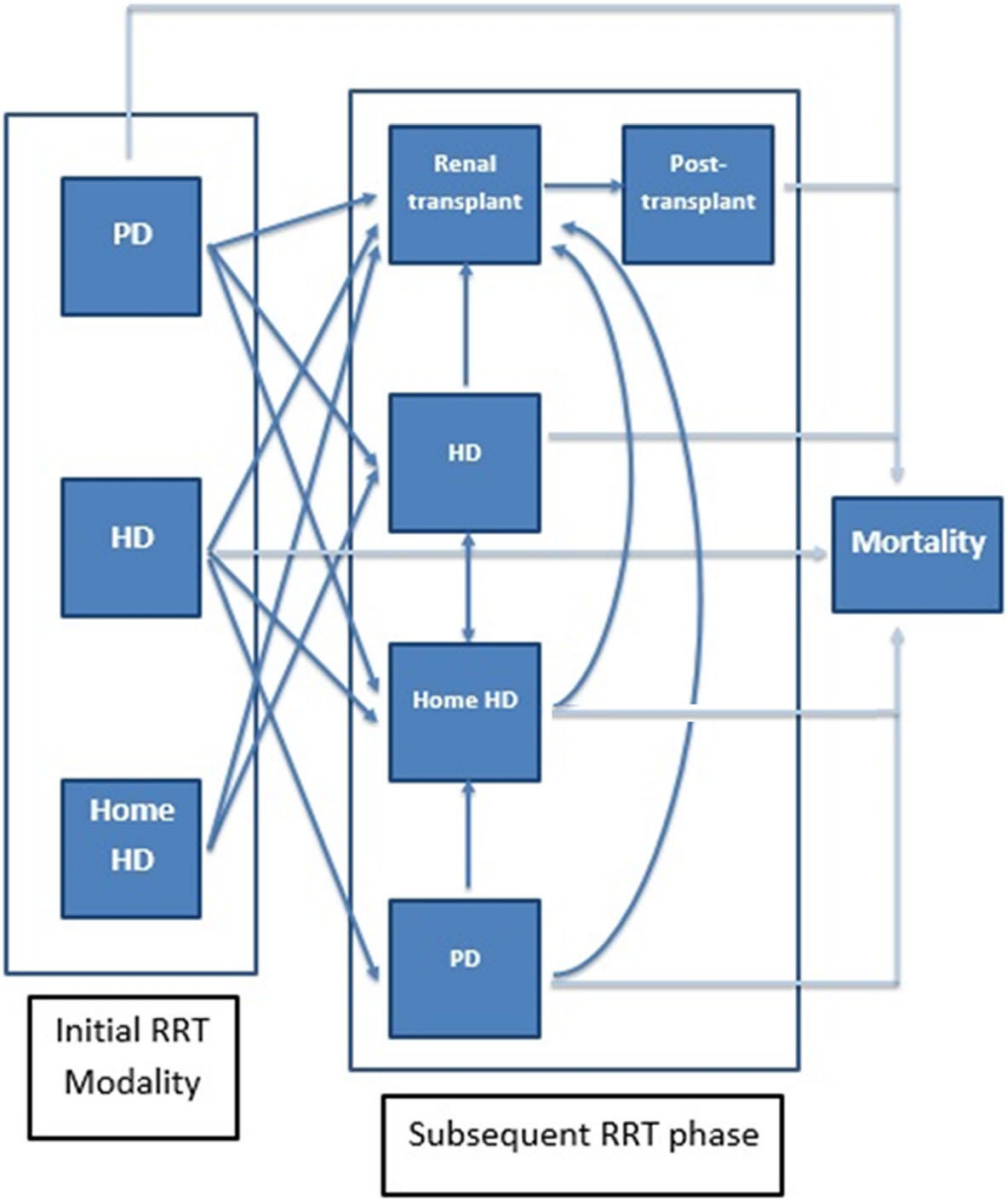
Annual transition between health states in lifetime simulation

Three first-line treatment strategies for ESRD patients, i.e. hospital-based HD, home-based HD and PD, were compared in terms of cost and effectiveness using a decision analytic model based on Markov modelling. A hypothetical static cohort of 100,000 individuals requiring RRT was entered into Markov modelling and simulated at age 60 (median age of ESRD entering into RRT was 59.1 in 2013 [5]) until the terminal states of death by 1-year cycle. Discounting at an annual rate of 3% was performed to annual total costs and QALYs before aggregation. The five principal Markov states of ESRD are “Home-based HD”, “Hospital-based HD”, “PD”, “Transplant and post-transplant”, and “Death”. To start with, each patient received one of the three initial RRT modalities. For each 1-year cycle, each patient stayed either at the same Markov state or at the transition to a new state based on given transition probabilities. Our model enabled the annual switching among three forms of dialysis, except for the one from home-based HD to PD. Renal transplanted patients would stay at post-transplant health state, and were prohibited from switching to any forms of dialysis. Annual mortality rates depend on current treatment modality, but independent of previous or initial treatment modalities if a change in treatment modality occurs. Patients undergoing home-based HD are not at risk of mortality because patients who develop complications are always admitted to hospital and are switched to hospital-based HD before they died. The annual health status transition between different health states and mortalities in Markov modelling is shown in Fig. 1. A summary of the assumptions on the model structure, health status transitions and parameters is presented in Table 2.

**Table 2:** Summary list of assumptions on the model structure, transition and parameters

Model Structure	
Health Status Transitions within one-year cycle	
• There are five principal health status: ‘PD’, ‘hospital-based HD’, ‘home-based HD’, ‘transplant and post-transplant’, and ‘death’	
• ESRD patients commencing home-based HD are prohibited from switching treatment modality to PD as there were no observed data / documented data from literature / data source	
• Post-transplanted patients are prohibited from modality switching to any forms of dialysis	
• Renal transplantation rates are identical for ESRD patients on any forms of dialysis.	
• Annual mortality rates depend on current treatment modality, but independent of previous or initial treatment modalities if the change in treatment modality occur	
Model Parameters	
Healthcare resource use and cost	
• Costs associated with each treatment modality (PD, hospital-based HD, home-based HD, transplant) are divided into initial year (or first year) and subsequent years (retaining the same treatment modality for 2 years or above)	
• Cost of pre-dialysis surgery accrues when patient initiates new dialysis	
• Cost of the surgery related to the removal of catheter / vascular access accrues when patient switches to another dialysis or undergoes renal transplantation	
• No removal of vascular access when patient switches from hospital-based HD to home-based HD and vice versa	
• No removal of catheter / vascular access when patient switches from any treatment modality to death	
• Dialysis complication is reflected in the number of outpatient visits, emergency visits, and the number of hospitalization days	
• Costs associated with renal transplantation and pre-dialysis surgery refer to the list of private charges to cost item in Hospital Authority. The charge covers surgeon fee, administration of anaesthetics, medicines used in operation, and operating theatre expenses	
• Cost of chronic haemodialysis, rather than acute haemodialys, is charged to each session of hospital-based HD	
• Unit costs of cadaveric and living-related transplantations, and transplantations performed outside HK are the same	
Health utility	
• Utility decrements depends on the current treatment modality. Utility decrements due to age, duration of ESRD, and duration of dialysis initiation are not taken into account	

Both the deterministic and probabilistic sensitivity analyses were performed to test the uncertainty and robustness of the results to changes in the cost and clinical parameters. Deterministic sensitivity analysis examined the impact of changing each specific parameter on modelling outputs such as mean incremental cost and effectiveness. Probabilistic sensitivity analysis was conducted to achieve a full examination of uncertainly involved in all parameters. All the model parameters except mortality rate were considered in sensitivity analyses because mortality rates for health status were estimated based on empirical data from Hong Kong Renal Registry [3, 5, 22]. Annual healthcare and societal costs in the first and second years of three dialysis groups reported from our previous cost analysis [13] were source of cost parameters for the first and subsequent years of three dialysis groups. Model parameters including cost, disutility and clinical parameters, alongside the range and simulated distribution for sensitivity analyses are listed in Table 3.

**Table 3:** Parameter values, ranges (95% confidence interval) and distributions used in the base-case scenario

Model parameter	Mean	SD	95% CI		Distribution	Parameter			Reference
			lower	upper					
Clinical									
Renal transplantation rate	0.0794								Leung 2015 [5]
Mortality rate (cases/100 person-years)									
PD	15.21								Ho 2013 [22]
Hospital-based HD	19.45								Ho 2013 [22]
Home-based HD	10.6								Rydell 2019 [23]
Post-transplant patients	2.00								Ho 2013 [22]
Annual probability of modality switching					Dirichlet				
PD →						159	19	1	
PD	0.888								Data from cost analysis
Hospital-based HD	0.106								Data from cost analysis
Home-based HD	0.006								Data from cost analysis
Hospital-based HD →						131	26	10	
Hospital-based HD	0.784								Data from cost analysis
PD	0.156								Data from cost analysis
Home-based HD	0.060								Data from cost analysis
Home-based HD →					Beta	(alpha,	beta)		
Home-based HD	0.950					41	2		McFarlane 2006 [21]
Hospital-based HD	0.050								McFarlane 2006 [21]
PD	0.000								assumption
Disutility					Gamma	(alpha,	beta)		
PD	0.222	0.110	0.200	0.243		4.06	0.055		Chen 2016 [17]
Hospital-based HD	0.269	0.114	0.249	0.288		5.58	0.048		Chen 2016 [17]
Home-based HD	0.222	0.091	0.194	0.250		5.94	0.037		Wong 2019 [18]
Transplant and post-transplant	0.190	0.492	0.100	0.280		17.12	0.011		Liem 2008 [16]
Annual costs									
Societal perspective					Gamma	(alpha,	beta)		
Total annual cost in initial year (USD)									
PD	24,255.3	7914.7	23,658.7	24,851.9		9.4	2582.7		Wong 2019 [13]
Hospital-based HD	57,968.0	9400.9	57,240.6	58,695.5		38.0	1524.6		Wong 2019 [13]
Home-based HD	31,030.6	3625.8	30,477.6	31,583.5		73.2	423.7		Wong 2019 [13]
Total annual cost in subsequent year (USD)									
PD	19,425.6	7737.6	18,842.4	20,008.9		6.3	3082.0		Wong 2019 [13]
Hospital-based HD	52,950.9	9423.3	52,221.7	53,680.1		31.6	1677.0		Wong 2019 [13]
Home-based HD	13,552.3	3058.0	13,085.9	14,018.6		19.6	690.0		Wong 2019 [13]
Healthcare provider perspective					Gamma	(alpha,	beta)		
Total annual cost in initial year (USD)									
PD	15,188.1	1994.8	15,037.8	15,338.5		58.0	262.0		Wong 2019 [13]
Hospital-based HD	51,289.4	8054.0	50,666.1	51,912.6		40.6	1264.7		Wong 2019 [13]
Home-based HD	28,635.7	2314.8	28,282.7	28,988.7		153.0	187.1		Wong 2019 [13]
Total annual cost in subsequent year (USD)									
PD	10,358.5	2028.2	10,205.6	10,511.4		26.1	397.1		Wong 2019 [13]
Hospital-based HD	46,272.2	8078.7	45,647.1	46,897.4		32.8	1410.5		Wong 2019 [13]
Home-based HD	11,157.4	1161.4	10,980.3	11,334.5		92.3	120.9		Wong 2019 [13]

*CI* Confidence Interval, *SD* Standard deviation;

The incremental cost-effectiveness ratio (ICER) was calculated by dividing the incremental cost by the incremental effectiveness in terms of QALYs gained for the hospital-based or home-based HD strategy relative to the reference PD strategy. A strategy that is more effective but less costly than the alternative was defined as a cost-saving strategy. A strategy was regarded as cost-effective relative to the alternative strategy if its incremental cost-effectiveness ratio is less than the threshold of one to three times gross domestic product per capita according to the cost-effectiveness threshold derived based on opportunity cost for Hong Kong SAR [24]. The Markov model was built using TreeAge Pro Suite (TreeAge Software, Williamstown, MA, USA).

## Results

### Base-case scenario

Table 4 shows the cost-effectiveness analysis over the lifetime horizon in base-case scenario from healthcare provider and societal perspectives. From the healthcare provider perspective, hospital-based HD group had higher cost USD$142,389 than PD but the lowest effectiveness 6.58 QALYs, and so it was dominated by the PD group (cost = USD$76,915; effectiveness = 7.13 QALYs). Home-based HD had the highest effectiveness 8.37 QALYs but with higher cost USD$97,917 than PD group. The ICER of USD$16,934 per QALY gained for home-based HD over PD. From the societal perspective, the results were similar to that of healthcare provider. The ICER was USD$1195 per QALY gained for home-based HD over PD. Both ICERs were less than thresholds derived based on opportunity costs.

**Table 4:** Lifetime cost (USD) of each dialysis modality strategy as first-line treatment and incremental cost-effectiveness ratio in base-case scenario

Perspective	Strategy	Cost (USD)	Incremental Cost (USD)	Effectiveness (QALY)	Incremental Effectiveness	ICER (USD / QALY gained)
Healthcare provider						
	PD^a^	76,915	NA	7.13	NA	NA
	Home-based HD	97,917	21,002	8.37	1.24	16,934
	Hospital-based HD	142,389	44,472	6.58	Dominated by PD	
Societal						
	PD^a^	109,668	NA	7.13	NA	NA
	Home-based HD	111,150	1482	8.37	1.24	1195
	Hospital-based HD	166,648	56,980	6.58	Dominated by PD	

*NA* Not applicable, *QALY* quality-adjusted life-year, *ICER* incremental cost-effectiveness ratio

^a^PD is the reference category when calculating incremental cost and incremental effectiveness

### Deterministic sensitivity analysis

**Fig. 2:**
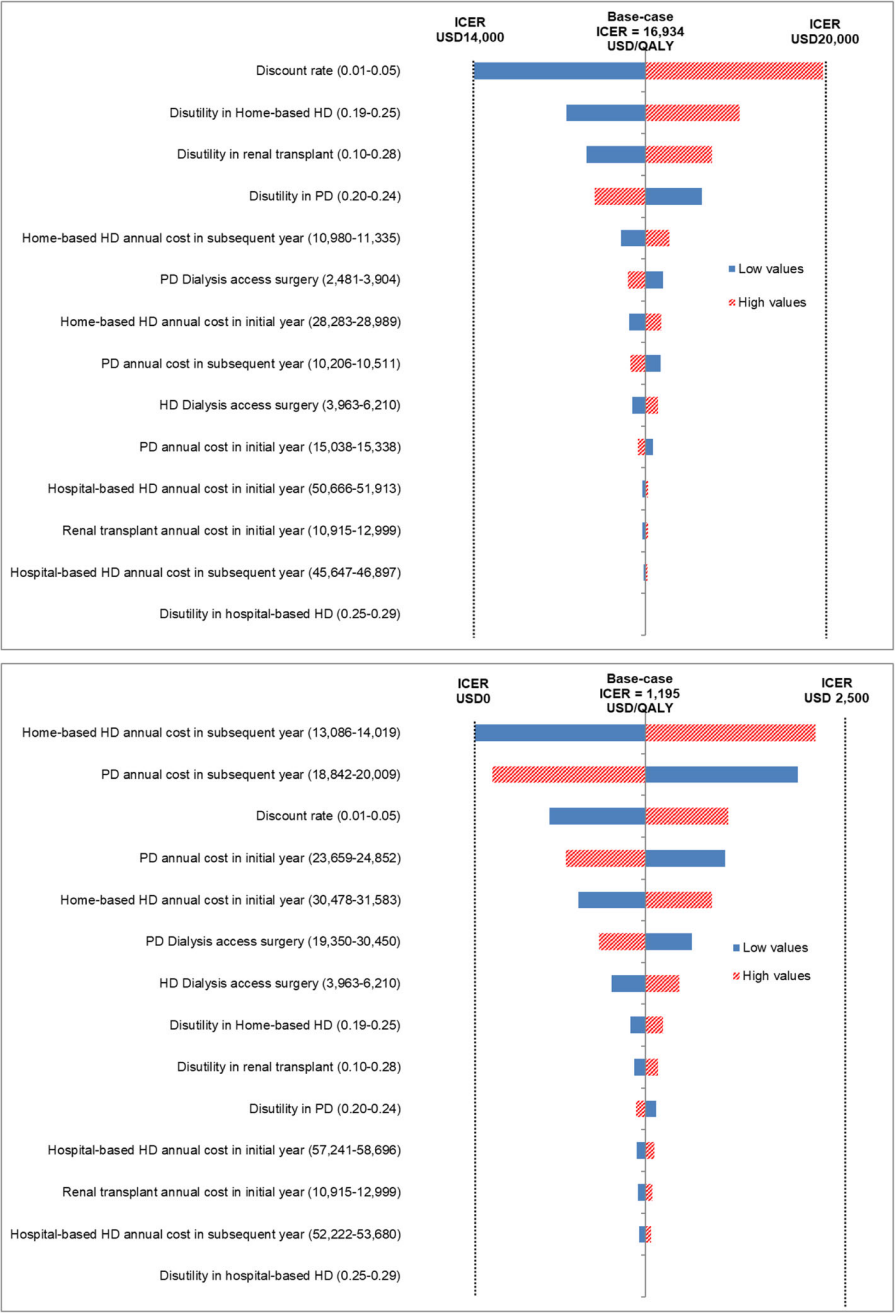
Tornado diagrams for one-way sensitivity analysis of incremental cost-effectiveness ratio of home-based haemodialysis relative to peritoneal dialysis **a**) from the healthcare provider perspective and **b**) from the societal perspective

The impact of changing each specific parameter on the cost-effectiveness results for the modality of Home-based HD versus PD from both the healthcare provider and societal perspectives are shown by tornado diagrams (Fig. 2a and b). Parameters that have the biggest impact are shown at the top of the tornado diagrams, whereas those that have the least impact are shown at the bottom. Both perspectives showed similar results that discount rate and disutility in renal transplant had the greatest impact while disutility in hospital-based HD and annual cost of hospital-based HD in initial year had the least impact on the cost-effectiveness. All model parameters had a minimal impact on ICER values.

### Probabilistic sensitivity analysis

**Fig. 3:**
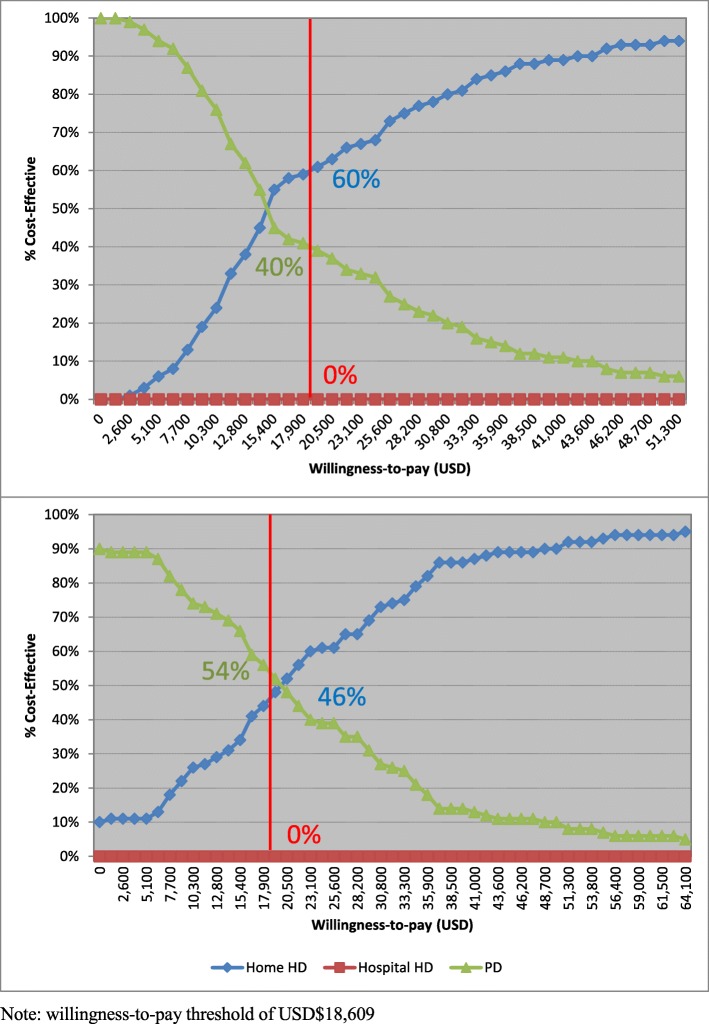
Cost-effectiveness acceptability curve **a**) from the healthcare provider perspective; and **b**) from the societal perspective

Cost-effectiveness acceptability curves (Fig. 3a and b) depicts that, from the perspective of healthcare provider, the probabilities of PD, hospital-based HD and home-based HD being the optimal treatment strategy were 40, 0, 60%, respectively, using an ICER threshold of USD$18,609 per QALY. Hospital-based HD was dominated at any ICER thresholds, and the probability of home-based HD being cost-effective was more than that of PD when the ICER threshold was higher than USD$18,609 and vice versa. The results from the societal perspective were similar to those from the healthcare provider perspective. The probabilities of PD, hospital-based HD and home-based HD being the optimal treatment strategy were 54, 0, 46%, respectively, using an ICER threshold of USD$18,609 per QALY. Hospital-based HD was dominated at any ICER thresholds, and the probability of home-based HD being cost-effective was more than that of PD when the ICER threshold was higher than USD$18,609 and vice versa.

## Discussions

Accumulative evidence by modelling studies demonstrated that renal transplantation was cost-saving and the most preferable strategy [10, 11, 25] but the opt-in consent system and scarcity of organ donations [6] limited the feasibility of renal transplantation as the first-line treatment modality in the management of ESRD patients. Cost-effectiveness analysis assessed the total costs and effectiveness for three competing strategies as first-line treatment, and identified the home-based HD strategy as the cost-effective first-line dialysis modality. Over a lifetime horizon based on a simpler cost-minimization approach, the PD strategy was the most preferable first-line dialysis strategy from both healthcare provider and societal perspectives. This finding echoed previous costing analyses based on the context of Hong Kong [2], Asian-Pacific countries [26] and other developed countries [25, 27].

By aggregating annual costs and effectiveness, hospital-based HD strategy accumulated the lowest total health effectiveness and high total costs, mainly attributable to expensive HD sessions in hospital setting. From both perspectives, PD strategy dominated hospital HD strategy. Our priori hypothesis that PD was more cost-effective than hospital-based HD from healthcare provider’s viewpoint was supported by our current finding. Such principal finding was in line with priority setting of other developed countries such as UK [12], Sweden [28], Singapore [29] and Taiwan [30], and thus supported the implementation of “Peritoneal Dialysis First” policy. When making pairwise comparisons between PD and home-based HD, the incremental cost-effectiveness ratio was USD$12,020 and USD$10,357 per one additional QALY gained from healthcare provider’s and societal viewpoints, respectively. Those ICER values were less than the upper bound that derived based on opportunity cost (range = USD$18,609-20,223) [24]. To interpret the cost-effectiveness of home-based strategy, the decision-making depends on whether the healthcare system and resources are willing to pay incremental costs for suitable ESRD patients for home-based HD. Current finding echoed the cost-utility analysis of hospital HD versus and home HD in the UK setting [31].

With respect to modelling aspects, our simulation model adopted one-year cycles in line with three previous modelling studies [10, 29, 32] but a shorter length of cycle such as monthly cycles [11, 12, 31] and 6-month cycles [28] was utilized in other published studies. This was in part due to the frequent occurrence of dialysis complications and modality switching within a short life expectancy for ESRD patients. By principle, the shorter the Markov cycle, the smaller is the error in the estimates of model outcomes [33], as simulation model is closer to the continuous time transition in reality. However, the choice of cycle length across simulation models reported consistent conclusions for cost-effectiveness comparison of PD versus hospital HD, capturing the cost and clinical advantages of PD strategy in comparison with hospital-based HD.

### Limitations

Our study has several limitations. First, the allocation of dialysis modality was not randomized due to the “Peritoneal Dialysis First” policy so demographic and clinical characteristics of patients varied across three dialysis modalities. Confounded by treatment indications, hospital-based HD patients were more medically complicated, incurring a higher treatment and complication costs [3], and thus might be clinically unfeasible to undergo home-based HD as first-line dialysis modality. Besides, continuous ambulatory peritoneal dialysis and automated peritoneal dialysis were subtypes of peritoneal dialysis, and were not classified as separate RRT strategy in in current analysis. Second, health utility scores applied in modelling were sourced from cross-sectional data primarily. Changes in health utility throughout the remaining lifetime and separation of utility scores between incident and prevalent ESRD patients were not taken into account in the cost-effectiveness model. Thirdly, the annual transition in our analysis relied on the assumption that renal transplantation rates were identical across three modalities, and post-transplant patients were prohibited from modality switch underlining the assumption that dialysis modality had no impact on renal graft. Finally, additional investments such as building capital and overhead costs associated with dialysis were identified in previous costing analyses [25, 32, 34, 35], and were not taken into consideration. Those elements were not possible to be quantified, and thus excluded from costs estimation in current analysis. Given in above, findings from this simulation models for healthcare setting under PD First policy might not be generalizable to healthcare setting in other countries.

## Conclusions

This cost-effectiveness analysis assessed lifetime costs of ESRD patients initiating hospital-based HD, home-based HD or PD dialysis modalities under the “Peritoneal Dialysis First” policy, and evaluated the cost-effectiveness of three dialysis modalities as first-line treatment from the health provider’s and societal viewpoints. Over a simulated lifetime horizon, hospital-based HD was dominated by PD from the healthcare provider perspective and societal perspective when indirect costs were taken into account. Those ICER values fell within threshold derived based on opportunity costs, supporting the PD First or favoured policy. Nocturnal home HD was the most effective strategy but more costly, which can be considered cost-effective for those patients who are suitable for home-based HD. Although there is no informed choice of dialysis under the “Peritoneal Dialysis First” policy, our results may help the general public, nephrologists and health policymakers to make recommendations and decisions about the management of ESRD patients.

## Data Availability

The data that support the findings of this study are available from database of the Clinical Management System of the Hospital Authority, Hong Kong but restrictions apply to the availability of these data, which were used under license for the current study, and so are not publicly available. Data are however available from the authors upon reasonable request and with permission of Hospital Authority, Hong Kong.

## Abbreviations

ESRD: End-stage renal disease
HD: Haemodialysis
Home HD: Nocturnal home HD
HRQOL: Health-related quality of life
ICER: Incremental cost-effectiveness ratio
PD: Peritoneal dialysis
QALY: Quality-adjusted life-year
RRT: Renal replacement therapies
SF-6D: Short-Form Six-Dimension
